# Expression and prognostic significance of TCTN1 in human glioblastoma

**DOI:** 10.1186/s12967-014-0288-9

**Published:** 2014-10-11

**Authors:** Delong Meng, Yuanyuan Chen, Yingjie Zhao, Jingkun Wang, Dapeng Yun, Song Yang, Juxiang Chen, Hongyan Chen, Daru Lu

**Affiliations:** State Key Laboratory of Genetic Engineering and MOE Key Laboratory of Contemporary Anthropology, School of Life Sciences, Fudan University, No. 2005 Songhu Road, Shanghai, 200438 People’s Republic of China; The Eighth Department of General Surgery and Department of Pathology, the First Affiliated Hospital of Anhui Medical University, No. 218 Jixi Road, Hefei, 230022 People’s Republic of China; Department of Neurosurgery, Shanghai Institute of Neurosurgery, Changzheng Hospital, Second Military Medical University, No. 415 Fengyang Road, Shanghai, 200003 People’s Republic of China

**Keywords:** TCTN1, Glioblastoma, Survival, Prognostic factor

## Abstract

**Background:**

Glioblastoma (GBM) is the most common and lethal intracranial malignancy in adults, with dismal prognosis despite multimodal therapies. Tectonic family member 1 (TCTN1) is a protein involved in a diverse range of developmental processes, yet its functions in GBM remain unclear. This study aims to investigate expression profile, prognostic value and effects of *TCTN1* gene in GBM.

**Methods:**

Protein levels of TCTN1 were assessed by immunohistochemical staining using a tissue microarray constructed by a Chinese cohort of GBM patients (n = 110), and its mRNA expression was also detected in a subset of this cohort. Kaplan-Meier analysis and Cox regression were performed to estimate the prognostic significance of TCTN1. Similar analyses were also conducted in another two independent cohorts: The Cancer Genome Atlas (TCGA) cohort (n = 528) and the Repository for Molecular Brain Neoplasia Data (REMBRANDT) cohort (n = 228). For the TCGA cohort, the relationships between *TCTN1* expression, clinical outcome, molecular subtypes and genetic alterations were also analysed. Furthermore, proliferation of TCTN1 overexpressed or silenced GBM cells was determined by CCK-8 assays.

**Results:**

As discovered in three independent cohorts, both mRNA and protein levels of TCTN1 expression were markedly elevated in human GBMs, and higher TCTN1 expression served as an independent prognostic factor predicting poorer prognosis of GBM patients. Additionally, in the TCGA cohort, *TCTN1* expression was dramatically decreased in patients within the proneural subtype compared to other subtypes, and significantly influenced by the status of several genetic aberrations such as *CDKN2A/B* deletion, *EGFR* amplification, *PTEN* deletion and *TP53* mutation. The prognostic value of *TCTN1* was more pronounced in proneural and mesenchymal subtypes, and was also affected by several genetic alterations particularly *PTEN* deletion. Furthermore, overexpression of TCTN1 significantly promoted proliferation of GBM cells, while its depletion evidently hampered cell growth.

**Conclusions:**

TCTN1 is elevated in human GBMs and predicts poor clinical outcome for GBM patients, which is associated with molecular subtypes and genetic features of GBMs. Additionally, TCTN1 expression impacts GBM cell proliferation. Our results suggest for the first time that TCTN1 may serve as a novel prognostic factor and a potential therapeutic target for GBM.

**Electronic supplementary material:**

The online version of this article (doi:10.1186/s12967-014-0288-9) contains supplementary material, which is available to authorized users.

## Background

Accounting for 45% of all brain malignancies and 54% of all human gliomas, glioblastoma (GBM) is the most aggressive and lethal type of brain tumor [1,2]. Despite multimodality therapies including maximal resection and adjuvant chemotherapy and radiotherapy, the overall outcome of patients with newly diagnosed GBM remains dismal. According to the most recent report of The Central Brain Tumor Registry of the United States (CBTRUS), less than 5% of GBM patients survive five years post diagnosis [1]. Clearly more effective therapies are urgently needed and identification of valuable prognostic biomarkers and potential molecular targets is one key strategy to achieve this goal.

There are several different genetic alterations of important genes that may contribute to the pathogenesis of GBM, and these aberrations may differ from patient to patient. Therefore, treatment regimens for patients with GBM may be more effective if they are tailored toward the particular pathogenesis of patients’ neoplasm. In recent years, substantial efforts have been made to explore molecular profiles to better understand the pathogenesis of GBM and biomarkers associated with patients’ survival. There also have been several public resources that have provided insight into the pathogenesis of GBM through allowing researchers to correlate levels of gene expression with clinical features, including The Cancer Genome Atlas (TCGA) network [3] and Repository of Molecular Brain Neoplasia Data (REMBRANDT) database [4]. Gene expression studies of TCGA GBM tissues have identified several distinct GBM molecular subtypes, namely classical, mesenchymal, proneural and neural [5]. Thus, uncovering new prognostic factors and molecular targets altered in GBM, and revealing the association of their expression profile with genetic alterations and molecular subtypes of GBM, may provide opportunities to improve the clinical outcome of GBM patients.

Tectonic family member 1 (TCTN1), was first identified in 2006 as a potential regulator of the Hedgehog pathway in patterning of the neural tube of mice, downstream of smoothened and Rab23, and named tectonic after the Greek word for builder due to its apparent involvement in a diverse range of developmental processes [6]. In addition, a recent study showed that TCTN1 was part of a ciliopathy-associated protein complex and interacted with several other proteins associated with ciliopathies [7]. Over the past several years, the primary cilium was found to be a complex signalling center where Hedgehog signalling was regulated [8-10], and its disregulation was associated closely to tumorigenesis [11-13]. Furthermore, Hedgehog pathway was involved in the regulation of embryonic development, cancer formation and maintenance, cancer stem cells [14-16], and particularly development and progression of human gliomas [17,18]. However, the function and prognostic value of TCTN1 in human glioma have never been characterized.

In this study, we sought to investigate levels of TCTN1 expression in human GBMs using a tissue microarray (TMA) of a Chinese GBM cohort and estimate its prognostic value. We then validated the differential expression and prognostic significance of *TCTN1* in another two independent datasets, namely the TCGA cohort and the REMBRANDT cohort. For the TCGA cohort, we also analysed the expression profile of *TCTN1* according to subtypes and genetic alterations of GBM. Finally, we performed cell proliferation assays to explore the functions of TCTN1 in GBM cells.

## Methods

### Patients and tissue samples

For the Chinese cohort of human glioblastoma (GBM) patients in this study, 110 specimens were obtained at the time of surgery at the Department of Neurosurgery in Changzheng Hospital, Second Military Medical University (SMMU), between January 2000 and December 2010. Tissues of 16 normal brain samples were taken from trauma outpatients. Clinicopathological information (age, gender, clinical manifestations and extent of resection) was obtained from medical records of the patients. Tumor histology was confirmed independently by two neuropathologists. Written informed consent was provided by all participants. The study protocol and acquisition of tissue specimens were approved by the Specialty Committee on Ethics of Biomedical Research, SMMU, Shanghai, China.

### Tissue microarray construction and immunohistochemistry

Formalin-fixed, paraffin-embedded tissues were used to construct an tissue microarray (TMA) as described previously [19,20] (Shanghai Biochip Company). Briefly, after verification with hematoxylin and eosin (H&E) staining, 1.5 mm core punch sample was taken from each specimen and cut as 4-μm-thick sections, which were then deparaffinized. Endogenous HRP activity was blocked with 3% H_2_O_2_, and antigen retrieval was achieved by boiling in sodium citrate buffer (pH 6.0). After blocking in 10% normal goat serum, immunostaining was performed using a rabbit anti-TCTN1 antibody (ab105381; Abcam) at 1: 50 dilution. Finally, the visualization signal was developed with 3,3’-diaminobenzidine (DAB), and the slides were then counterstained in hematoxylin. As negative controls, the sections were incubated with normal mouse serum instead of the primary antibody. The scores of immunohistochemical staining were evaluated by two independent pathologists in a blinded manner as described previously [21-23]. Briefly, the expression of TCTN1 was scored by estimating the proportion of tumor cells with positive staining. High TCTN1 expression was defined as >10% positive staining, while low expression was defined as a proportion of < = 10% positive staining, as described in previous studies [22,24-31].

### RNA extraction, cDNA synthesis, and quantitative real-time PCR

Fresh-frozen tissues from 8 human GBM patients and 8 normal brain samples were used for total RNA extraction using the Trizol reagent (Invitrogen) according to the manufacturer’s instructions. Reverse transcription of total RNA was conducted using ReverTra Ace qPCR RT Master Mix (Toyobo), and quantitative real-time PCR was performed using THUNDERBIRD SYBR qPCR Mix (Toyobo) on ABI PRISM 7900HT instruments (Applied Biosystems). The primers used for amplification of TCTN1 were as follows: sense, 5’-CTGGATATTCCTACTGCTGCTAAAT-3’; antisense, 5’-CGAAGGAAATCTCAGAAACGA-3’. Glyceraldehyde-3-phosphate dehydrogenase (GAPDH) was used as the endogenous control, using primers: sense, 5’-AGCCACATCGCTCAGACAC-3’; antisense, 5’-GCCCAATACGACCAAATCC-3’. The amplification was done in a total volume of 10 μl with the following conditions: an initial denaturation step (95°C for 5 minutes), followed by 40 cycles of denaturation (95°C for 15 seconds) and elongation (60°C for 45 seconds), and a melting curve analysis of each sample was used to check the specificity of amplification. Each sample was assayed in triplicate, and the 2^-ΔΔCt^ method [32] was used to determine relative gene expression.

### *In silico* analyses of TCGA and REMBRANDT data

Another two independent datasets of GBMs, The Cancer Genome Atlas (TCGA) [3] cohort (n = 528) and the Repository of Molecular Brain Neoplasia Data (REMBRANDT) [4] cohort (n = 228), were also included in the present study. Expression data of *TCTN1* and clinical information of patients were obtained to validate the differential expression of *TCTN1* and its prognostic value. For the TCGA cohort, we also obtained common mutations, copy number alterations and molecular subtypes data, which were available for part of the patients, to analyze the expression profile of *TCTN1* and its relationship with these items. In the analysis of TCGA cohort, TCTN1 levels were dichotomized to high and low at the median expression as previously described [33-35]. For the REMBRANDT cohort, analyses were performed on the website interface using the default parameters [23,36].

### Cell culture

U251 and U87 human GBM cell lines, and 293 T human embryonic kidney cell line were purchased from Cell Bank of Chinese Academy of Sciences (Shanghai, China), and cultured in Dulbecco’s modified Eagle’s medium (DMEM; Life Technologies) supplemented with 10% fetal bovine serum (FBS; Life Technologies) and penicillin/streptomycin (100 units/ml and 100 μg/ml, respectively; Life Technologies) and maintained at 37°C in an atmosphere of humidified air containing 5% CO_2_.

### Gene overexpression and silencing

To overexpress TCTN1, coding sequence of *TCTN1* gene was cloned into a lentiviral vector pCDH-CMV-EF1-copGFP (pCDH; System Biosciences) at the *Xho*I and *Eco*RI restriction sites using primers: sense, 5’-CCGCTCGAGACTCCCTGGGAGATGAGGC-3’; antisense, 5’-GGAATTCTCAAACAAACGGGAAGAAGAAG-3’. To interfere TCTN1 expression, the 21-nucleotide target sequence was selected from the Public TRC Portal [37]: shTCTN1(clone ID, TRCN0000297995), 5’-CTTCAGATTCGTTTCTGAGAT-3’. Sequence against LacZ gene served as a control designated shControl: 5’-GGATCAGTCGCTGATTAAA-3’ [38]. Corresponding sense and antisense oligonucleotides were synthesized, annealed and cloned into the *Hpa*I - *Xho*I sites of pLL3.7 lentiviral vector [39]. Lentiviral production and transduction was conducted as previously described [40]. Briefly, 293 T cells were co-transfected with the lentiviral expression vector pCDH-TCTN1 (pCDH empty vector as a control) together with packaging plasmids pLP/VSVG, pLP1 and pLP2 for overexpression, and with lentiviral vector pLL3.7-shTCTN1 (or shControl) and corresponding packaging vectors psPAX2 and pMD2.G for gene silencing, using Lipofectamine 2000 (Invitrogen) according to the manufacturer’s instructions. The supernatants of lentiviral particles were collected 48 hours post transfection and filtered through 0.45-μm syringe filters (Millipore). U251 and U87 cells were infected with the lentiviruses carrying the expression vector or shRNA against TCTN1 along with corresponding controls.

### Cell proliferation assay

Cell proliferation assay was performed as previously described [41]. Briefly, different cell lines were seeded in 96-well plates (1500–2000 cells/well) in six replicates. Cells were allowed to grow for 4 days and cell proliferation analysis was performed by Cell Counting Kit-8 (CCK-8; Dojindo Laboratories) assay at different time points according to the manufacturer’s instructions. After an incubation of 2 hours at 37°C, absorbance was measured at 450 nm using a microplate reader iMark (Bio-Rad).

### Western blot

Western blot was performed as previously described [42]. Briefly, cells were lysed in the radioimmunoprecipitation assay (RIPA) buffer [50 mM Tris–HCl pH 8.0, 150 mM NaCl, 1% (v/v) NP-40, 0.5% (w/v) Sodium deoxycholate, 0.1% (w/v) SDS] with protease inhibitors cocktail (Sigma) added freshly. The lysates were separated by 10% SDS-PAGE and transferred to polyvinylidene difluoride membranes (Millipore), which were blocked in 5% milk for 1 hour and then probed with antibody against TCTN1(1:200; ab105381; Abcam), or actin (1:4000; M20010; Abmart) as a loading control. Blots were developed with Immobilon Western Chemiluminescent HRP Substrate (Millipore) and visualized on G: Box Chemi XR5 (Syngene).

### Statistical analysis

Differences between two groups were analyzed by two-tailed student’s t-test. The Fisher’s exact test (two-sided) was conducted to analyze the correlation between *TCTN1* expression and clinical characteristics. Pearson correlation test was performed to analyze the correlation between expression of *TCTN1* and other genes. Overall survival (OS) was defined as the elapsed time between diagnosis and death or the last follow-up, and progression-free survival (PFS) was defined as the time from diagnosis to the date of tumor recurrence or further growth of residual tumor or the date of death. Survival curves were plotted by the Kaplan-Meier method and compared by the log-rank test. To construct a model for the prediction of survival, univariate and multivariate Cox proportional-hazards regression analysis was performed, in which clinical variables with log-rank *P* <0.05 in univariate analysis were pooled into multivariate analysis. Values presented are expressed as mean ± SD. SPSS (15.0) software (SPSS Inc.) was used for all statistical analysis and *P* <0.05 was considered statistically significant.

## Results

### GBM tissues exhibited increased protein and mRNA expression of *TCTN1* gene

**Figure 1:**
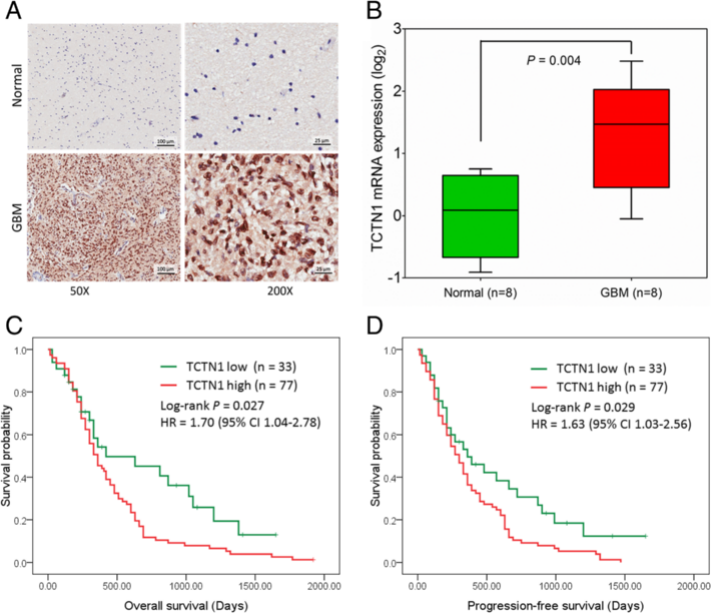
**Expression and prognostic value of TCTN1 in a Chinese glioblastoma (GBM) cohort. (A)** TCTN1 protein expression was analysed by immunohistochemistry staining in human GBM specimens and normal brain samples, and representative images show high nuclear expression of TCTN1 in GBMs. Magnification: ×50, left; ×200, right. Scale bars: 100 μm, left; 25 μm, right. **(B)** *TCTN1* mRNA expression was analysed by real time RT-PCR assay in human GBM samples, and *GAPDH* was used as an internal control. *P* value was determined by Student’s t test. **(C-D)** Kaplan-Meier plots were estimated according to different TCTN1 immunoreactivity level for overall survival **(C)** and progression-free survival **(D)** of GBM patients. *P* values were obtained from log-rank test, and hazard ratio (HR) and 95% confidence interval (CI) were calculated by univariate Cox regression model.

In total, 110 cases of GBM patients were enrolled in the Chinese GBM cohort. The median age at diagnosis was 53 years. Of the subjects, 74 (67.3%) were males. Details of clinical characteristics were presented in Table 1. The patients’ median overall survival (OS) was 12 months, with 5 year survival rate of 4.3%. We assessed the protein expression of TCTN1 in 110 GBMs and 16 normal brain tissues by immunohistochemistry assay using a tissue microarray (TMA). We found that TCTN1 was mainly expressed in nucleus (Figure 1A), and was significantly increased in GBM tissues compared to normal controls (*P* =0.042, Additional file 1: Figure S1), with no significant correlation with gender, age and other clinicopathologic characteristics. We further addressed whether *TCTN1* gene was also up-regulated at the transcriptional level. Total RNA was extracted from a subset of 8 GBMs and 8 controls randomly selected from this cohort and subjected to real-time quantitative RT-PCR assay. The mRNA expression of *TCTN1* was considerably elevated in GBMs compared to normal controls (Figure 1B, *P* =0.004).

**Table 1:** **Correlation between TCTN1 expression and clinicopathologic characteristics of 110 GBM patients in the Chinese cohort**

**Characteristics**	**No. patients**	**TCTN1 expression**		***P*** *
		**Low**	**High**	
Gender				0.378
Male	74(67.3%)	20(27.0%)	54(73.0%)	
Female	36(32.7%)	13(36.1%)	23(63.9%)	
Age (year)				0.211
≥53	52(47.3%)	19(36.5%)	33(63.5%)	
<53	58(52.7%)	14(24.1%)	44(75.9%)	
Tumor origin				0.670
Primary	95(86.4%)	28(29.5%)	67(70.5%)	
Secondary	15(13.6%)	5(33.3%)	10(66.7%)	
Seizure				0.349
No	96(87.3%)	27(28.1%)	69(71.9%)	
Yes	14(12.7%)	6(42.9%)	8(57.1%)	
IICP				0.836
No	59(53.6%)	17(28.8%)	42(71.2%)	
Yes	51(46.4%)	16(31.4%)	35(68.6%)	
Cystic degeneration				0.799
No	82(74.5%)	25(30.5%)	57(69.5%)	
Yes	23(20.9%)	6(26.1%)	17(73.9%)	
Necrosis				0.419
No	90(81.8%)	25(27.8%)	65(72.2%)	
Yes	20(18.2%)	8(40.0%)	12(60.0%)	
Edge				0.762
Not clear	52(47.3%)	15(28.8%)	37(71.2%)	
Clear	17(15.5%)	6(35.3%)	11(64.7%)	
MTD (cm)				0.093
< 5	46(41.8%)	18(39.1%)	28(60.9%)	
≥5	64(58.2%)	15(23.4%)	49(76.6%)	
Resection				1.000
≥98%	84(76.4%)	25(29.8%)	59(70.2%)	
< 98%	26(23.6%)	8(30.8%)	18(69.2%)	

*P value was evaluated by Fisher's Exact Test (2-sided).

*Abbreviations*: IICP, increased intracranial pressure; MTD, mean tumor diameter.

### TCTN1 served as an independent prognostic factor for GBM patients

To investigate the correlation between TCTN1 expression and clinical outcome, we first analysed the prognostic significance of TCTN1 using Kaplan-Meier method. As shown in Figure 1C and D, high TCTN1 expressers had significantly shorter overall survival (OS) and progression-free survival (PFS) than those with low TCTN1 expression (Log rank *P* =0.027 and 0.029 for OS and PFS respectively). Moreover, the subsequent univariate Cox regression indicated that, besides TCTN1 expression (HR =1.70, 95% CI =1.04-2.78, *P* = 0.033 for OS; HR = 1.63, 95% CI =1.03-2.56, *P* =0.036 for PFS), age at diagnosis was also a significant prognostic factor. As shown in Table 2, multivariate Cox regression revealed that, after correction for patient age, elevated expression of TCTN1 protein was an independent risk predictor of both OS (HR =1.69, 95% CI =1.03-2.76, *P* =0.037) and PFS (HR =1.60, 95% CI =1.01-2.52, *P* =0.044) for GBM patients in the Chinese cohort.

**Table 2:** **Multivariate Cox regression analysis of** ***TCTN1*** **expression in GBM patients of 3 independent cohorts**

**Cohort**	**Characteristics**	**Multivariate cox regression**	
		**HR (95% CI)**	***P***
Chinese GBM (OS)	*TCTN1* (high vs. low)	1.69 (1.03-2.76)	0.037
	Age (≥53 vs. <53)	1.54 (1.02-2.33)	0.038
(PFS)	*TCTN1* (high vs. low)	1.60 (1.01-2.52)	0.044
	Age (≥53 vs. <53)	1.46 (0.98-2.18)	0.061
TCGA (OS)	*TCTN1* (high vs. low)	1.26 (1.03-1.54)	0.026
	Age (≥60 vs. <60)	1.90 (1.55-2.33)	<0.001
REMBRANDT (OS)	*TCTN1* (high vs. low)	1.58 (1.09-2.29)	0.017
	Age (≥60 vs. <60)	2.23 (1.57-3.16)	<0.001

*Abbreviations*: OS, overall survival; PFS, progression-free survival; CI, confidence interval; HR, hazard ratio.

### *TCTN1* gene was overexpressed in GBMs and correlated with several clinical features in the TCGA cohort

**Figure 2:**
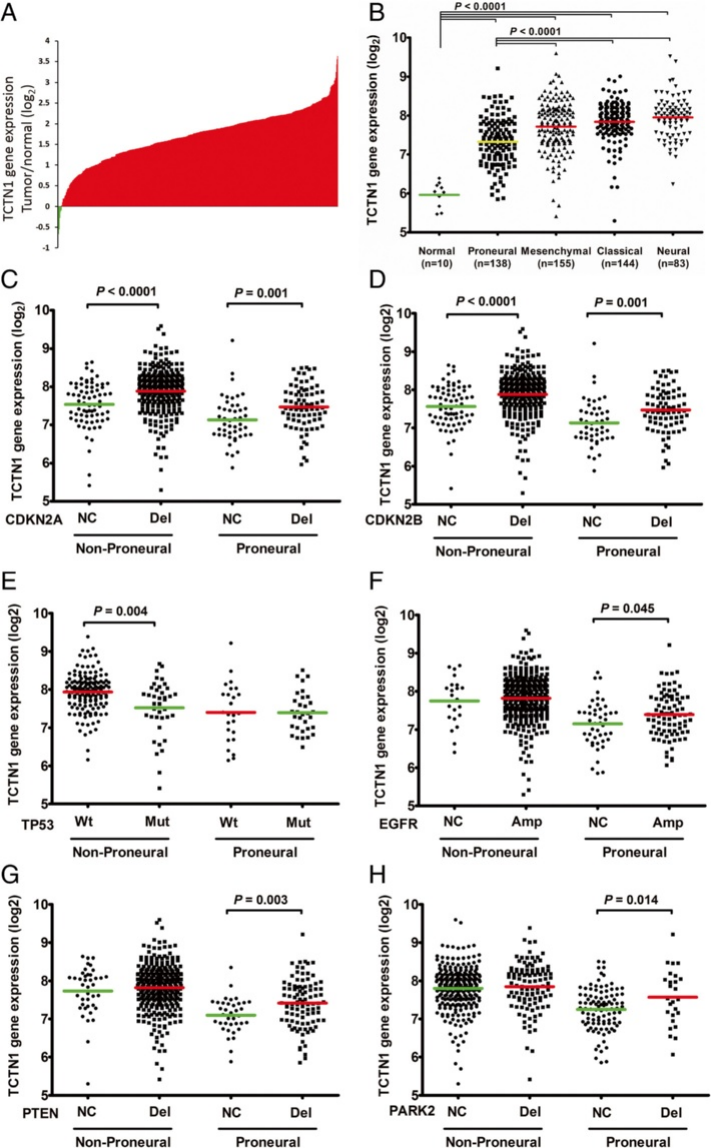
**Expression of** ***TCTN1*** **mRNA in GBM specimens of the TCGA cohort. (A)** *TCTN1* mRNA expression levels were detected in 528 clinical GBM specimens and 10 cases of normal control tissue obtained by TCGA. The value represents log 2 of gene expression value of GBM to the average mRNA of 10 normal samples. The red samples (>0) indicate that the mRNA levels of these GBM tissues were higher than the average of normal brain tissues while the green bars (<0) represent GBM sample with lower *TCTN1* mRNA expression compared to normal tissues. **(B-H)** *TCTN1* mRNA expression was significantly different in subgroups of GBM according to subtypes and/or status of common mutations or copy number alterations (CNA) as indicated. “NC”, no change; “Del”, deletion; “Amp”, amplification; “Wt”, wild-type; “Mut”, mutation. A single spot represents the *TCTN1* expression value (log 2 scale) of an individual patient, with a line in the middle representing the mean expression value. The difference in *TCTN1* expression was determined by Student’s t-test.

We next examined the expression profile and clinical significance of *TCTN1* in an independent cohort, i.e. the TCGA cohort. Consequently, mRNA expression of *TCTN1* was found to be increased in 98.86% (522/528) of the GBMs compared to the normal brain controls (Figure 2A). TCGA network described a robust gene expression-based molecular classification of GBM into 4 different subtypes, namely classical, mesenchymal, neural, and proneural [5]. Thus, we further screened *TCTN1* expression in different molecular subtypes of GBMs and found that *TCTN1* expression was dramatically decreased in proneural subtype compared with other three subtypes, although still significantly elevated as compared to normal controls (*P* <0.0001; Figure 2B).

In TCGA analysis of GBM, several genes were identified to be significantly mutated or have significant copy number alterations (CNAs) [3,43]. To further explore the expression profile of *TCTN1* gene, we examined associations between its expression and common genetic alterations in GBM, including mutations in *TP53*, *PTEN*, *NF1*, *EGFR*, *RB1*, *PIK3R1*, *IDH1*, *PIK3CA*, *SPTA1*, *ATRX*, *KEL*, *GABRA6*, *LZTR1*, *CTNND2*, *BRAF*, amplifications of *EGFR*, *CDK4*, *PDGFRA*, *MDM2*, *MET*, *MDM4*, *CDK6*, *MYCN*, *CCND2*, *PIK3CA*, *AKT3*, and deletions of *CDKN2A*, *CDKN2B*, *PTEN*, *CDKN2C*, *RB1*, *PARK2* and *NF1*. Consequently, we found that *TCTN1* expression was significantly associated with mutations of *TP53*, *IDH1* and *ATRX*, amplifications of *EGFR*, *PDGFRA* and *MYCN*, and deletions of *CDKN2A*, *CDKN2B*, *PTEN* and *PARK2* (Figure 2C-H, Additional file 1: Figure S2), but not other aberrations.

Given that *TCTN1* expression was also correlated with transcriptional subtypes, we next addressed whether the differential expression of *TCTN1* according to abovementioned genetic alterations was dependent on specific subtypes. As a result, *CDKN2A* or *CDKN2B* deleted cases had significantly higher expression of TCTN1 in both non-proneural (*P* <0.0001) and proneural (*P* =0.001) subtypes compared with cases with no corresponding changes (Figure 2C-D). Cases with *TP53* mutation had lower levels of *TCTN1* expression than wild-type (*P* =0.004) in non-proneural subtypes, while there was no significant difference in proneural subtype (Figure 2E). In contrast, cases with amplification of *EGFR* or deletion of *PTEN* or *PARK2* had higher levels of *TCTN1* expression than cases with no corresponding changes only in proneural subtype (*P* =0.045, 0.003, 0.014, respectively; Figure 2F-H). However, the differential expression of *TCTN1* according to mutations of *IDH1*, *ATRX* or amplification of *MYCN* and *PDGFRA* (Additional file 1: Figure S1A-D) lost any statistical significance when we classified all cases into non-proneural and proneural subgroups.

Given that previous studies linked TCTN1 to Hedgehog pathway in mouse embryonic development [6], we investigated the associations between the expression level of *TCTN1* and common targets of Hedgehog pathway, *GLI1* and *PTCH1*, and found no significantly correlation (Additional file 1: Figure S3).

### *TCTN1* was associated with prognosis of GBM patients in the TCGA cohort

**Figure 3:**
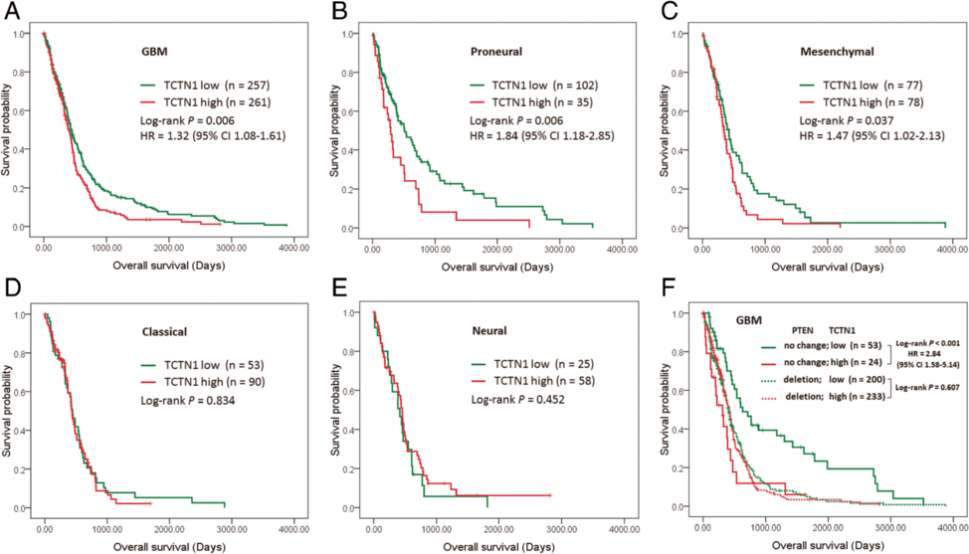
**The prognostic value of** ***TCTN1*** **in GBM specimens of the TCGA cohort.** Kaplan-Meier plots were estimated according to different *TCTN1* gene expression for overall survival of all GBM patients **(A)** or the 4 different subtypes of GBM patients **(B-E)**, or considering the copy number alteration status of *PTEN* simultaneously **(F)**. *P* values were obtained from log-rank test, while hazard ratio (HR) and 95% confidence interval (CI) were determined by univariate Cox regression model.

We further investigated the relationship between *TCTN1* expression and patients’ clinical outcome in the TCGA cohort. We compared the survival of all GBM patients with *TCTN1* expression above or below the median expression and found a statistically significant disadvantage in overall survival for patients with higher *TCTN1* expression (Log-rank *P* =0.006; Cox regression HR =1.32, 95% CI 1.08-1.61; Figure 3A). Multivariate Cox regression further confirmed the prognostic value of *TCTN1* as an independent predictor (HR =1.60, 95% CI =1.01-2.52, *P* =0.044; Table 2).

In addition, we also carried out survival analyses for each molecular subtype and found that only the proneural (Log-rank *P* =0.006; Cox regression HR =1.84, 95% CI 1.18-2.85) and mesenchymal (Log-rank *P* =0.037; Cox regression HR =1.47, 95% CI 1.02-2.13) subtypes retained statistical significance (Figure 3B-E).

Furthermore, we performed survival analysis stratified by the genetic alteration status of abovementioned 10 genes with which *TCTN1* expression was significantly associated. As a consequence, *TCTN1* expression was associated with patients’ prognosis only in one specific subgroup classified by the status of 7 genes (*PTEN*, *EGFR*, *PDGFRA*, *MYCN*, *PARK2*, *CDKN2A*, *CDKN2B*; Figure 3F and Additional file 1: Figure S4). A representative example shown in Figure 3F indicated that prognostic significance of *TCTN1* was highly pronounced in individuals with no *PTEN* change (Log-rank *P* <0.001; Cox regression HR =2.84, 95% CI 1.58-5.14), but not significant in *PTEN* deleted individuals. However, for the other 3 genes, namely *TP53*, *IDH1* and *ATRX*, *TCTN1* expression could not predict patients’ outcome in any subgroup stratified by the genetic status of these genes.

### The differential expression and prognostic value of *TCTN1* was further validated in the REMBRANDT cohort

**Figure 4:**
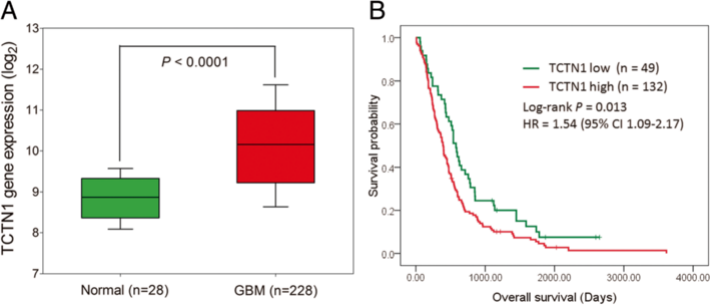
**Expression and prognostic value of** ***TCTN1*** **in GBMs of the Rembrandt cohort. (A)** *TCTN1* gene has significantly higher expression in GBM samples in comparison to non-tumor controls. *P* value was calculated by Student’s t-test. **(B)** Kaplan-Meier curve was plotted according to different *TCTN1* gene expression for overall survival of GBM patients. *P* values were obtained from log-rank test, while hazard ratio (HR) and 95% confidence interval (CI) were determined by univariate Cox regression model.

We further validated the differential expression and prognostic significance of *TCTN1* in GBMs of another independent cohort, namely the REMBRANDT cohort. Consistent with above mentioned TMA and TCGA analysis, *TCTN1* gene expression was remarkably increased in GBMs (n = 228) than in normal controls (n = 28; *P* <0.0001; Figure 4A). Moreover, high *TCTN1* mRNA expression (n =132) could significantly predict a worse overall survival for GBM patients in comparison with low *TCTN1* expression (n =49; Log-rank *P* =0.013; HR =1.54, 95% CI 1.09-2.17; Figure 4B), which could also serve as an independent prognostic factor in a multivariate Cox regression model (HR =1.58, 95% CI =1.09-2.29, *P* =0.017; Table 2).

### Ectopic TCTN1 expression affected GBM cell proliferation

**Figure 5:**
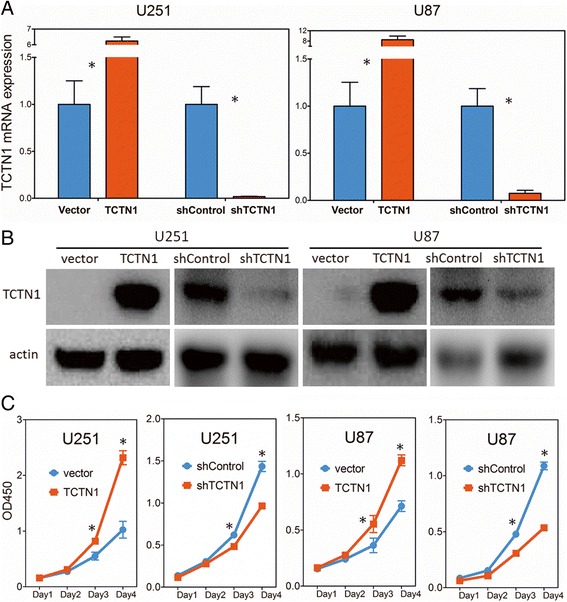
**Overexpression and knock-down of** ***TCTN1*** **regulate GBM cell growth. (A)** Overexpression and knock-down of *TCTN1* gene in U251 and U87 human GBM cell lines were validated at mRNA level by real-time RT-PCR assays and GAPDH was used as an internal control. **(B)** Protein level of TCTN1 was analysed by western blot assays and actin served as a loading control. **(C)** The cell growth curve of *TCTN1* overexpression and knockdown cells was assessed by CCK-8 assay. Each experiment was performed three times. Statistical analysis was performed using two tailed Student’s t-test. *, *P* < 0.05.

To explore the biological significance of TCTN1 in glioma, we investigated whether it could affect cell proliferation. TCTN1 was stably overexpressed or silenced in U251 and U87 cells by lentiviruses infection, while the efficiency of ectopic expression of TCTN1 was validated by real-time PCR (Figure 5A) and western blot (Figure 5B) analysis. We then studied the impact of TCTN1 on GBM cell proliferation by CCK-8 assay within a 4-day period monitoring. The results showed that in both U251 and U87 GBM cell lines, upregulation of TCTN1 significantly promoted the proliferation compared with the control groups, whereas the blockade of endogenous TCTN1 expression markedly inhibited cell growth in comparison with the controls (Figure 5C).

## Discussion

Glioblastoma (GBM) is the most malignant brain tumor with dismal prognosis despite multimodal therapies, and its pathogenesis is still far from elucidation. Molecular targeted therapy represents promising avenue for the future of effective treatment strategies for GBMs. Hence, more valuable prognostic biomarkers and potential molecular targets for gliomas are urgently needed to combat this devastating disease. The present study identified TCTN1 as a novel prognostic factor for GBM, which was overexpressed in GBM tissues and could also regulate the proliferation of GBM cells.

*TCTN1* was a newly identified gene reported to be involved in developmental processes, Hedgehog pathway transduction and functions of primary cilium [6,7]. Given that potent regulators of developmental processes are frequently disrupted in tumorigenesis [44], and the primary cilium and Hedgehog pathway also play important roles in tumorigenesis [11,16], it is to be expected that TCTN1 also contributes to tumor development yet there have been no reports on it. Hence, our study aimed to unveil the indispensable role of TCTN1 in GBM progression. Our TMA analysis and real-time PCR validation of a Chinese GBM cohort revealed that TCTN1 was up-regulated in GBMs compared to normal controls, and high TCTN1 expression could predict shorter overall and progression-free survival for GBM patients, as an independent prognostic factor. Due to differences of genetic background between populations [45], we further validated these findings in another two independent international cohorts, namely the TCGA cohort and the REMBRANDT cohort.

It was noteworthy that our immunohistochemical staining experiments in GBM tissues found a nuclear localisation of TCTN1, which was beyond our expectation more or less, given the two important reports that linked TCTN1 to Hedgehog pathway and primary cilia by Dr. Jeremy F. Reiter’ group [6,7]. Actually, there were several limitations of these studies. The former revealed the involvement of Tctn1 (the mouse homolog of human TCTN1) in Hedgehog signaling mediated patterning of the neural tube of mice. Epistasis analyses further indicated that Tctn1 modulated Hedgehog signal transduction downstream of Ptch, Smo and Rab23. However, the findings were merely restricted in a mouse embryonic development context and lacked direct evidences using biochemical methods. The latter report found that Tctn1 was essential for ciliogenesis in some embryonic tissues such as the node and neural tube, and was required to localize some proteins to the cilium in several other tissues containing perineural and limb bud mesenchyme. They further discovered Tctn1 as part of a transition zone complex that controlled the organization of the transition zone and ciliary membrane composition using some mouse cell lines. However, the mechanisms underlying the tissue specificity of Tctn1 complex function remain unclear and the findings were also context dependent. Thus, whether TCTN1 regulate Hedgehog pathway still remains unclear, particularly in the context of human cancer biology.

Hedgehog signaling pathway was linked to tumorigenesis in recent years. The most typical examples were basal cell carcinoma (BCC) [46] and medulloblastoma (MB) [47], in which mutations were identified in the regulatory components of Hedgehog pathway. Although there were a few reports regarding the regulation of Hedgehog signaling on cancer stem cells in human gliomas [48,49], the role of Hedgehog pathway in glioma remains in question.

To further investigate the relationship of *TCTN1* and Hedgehog pathway, we examined the transcriptional level of *GLI1*, which is widely used to reflect Hedgehog pathway activity [50], in TCGA database, and found it comparable between GBMs and normal controls (data not shown). In addition, we analyzed the relationship of *TCTN1* and two common target genes of Hedgehog pathway, *GLI1* and *PTCH1*, and found no significant correlation (Additional file 1: Figure S3), indicating a rather weak link (if any) between TCTN1 and Hedgehog pathway in GBMs.

The signal transduction of Hedgehog pathway was regulated in the primary cilium, where TCTN1 was found to be a component of a protein complex. In the mammalian body, primary cilia were found on most epithelial and stromal cells, and interestingly, transformed cells commonly lack cilia [51]. The role of primary cilia in cancer progression were still controversial, maybe according to the genetic background, as found in BCC [13] and MB [12]. In addition, the prevalence and role of cilia on glioma cells were poorly studied. It was reported that primary cilia were deficient in several established GBM cell lines compared to normal astrocytes [52]. Consistently, in recently derived primary GBM cell lines and tumor biopsies, the majority of cells were unable to grow cilia [53]. Furthermore, it seems that the observed cilia of a small portion of U251 GBM cells had no effect on cell proliferation, since depletion of Kif3a, a key component of ciliogenesis, did not significantly affect cell growth [54].

A remarkable feature of ciliogenesis is its cell cycle-dependence [51,55-57]. In a system to study ciliary dynamics in the hTERT-RPE1 cell lines, most of the cells were ciliated following serum starvation [56], which was widely used to induce ciliogenesis in cultured cells [54,58,59]. However, it is a remarkable fact that ciliogenesis was enhanced by serum starvation in neither established nor recently derived primary GBM cell lines [52,53], although that was the case in normal primary astrocytes [52]. Recently, it was reported that a cell-cycle-related kinase (CCRK) may modulate ciliogenesis, and its regulation of cell cycle was dependent on cilia in NIH3T3 cells [54]. In addition, they found that depletion of CCRK could restore cilia for a small fraction of U251 glioma cells, and inhibit cell growth in part dependent on cilia. However, it is interesting to note that depletion of CCRK did not block cells in G0/G1 phase, suggesting other underlying mechanisms.

Our immunohistochemical staining experiments showed primary expression of TCTN1 in cell nucleus through a scan of more than one hundred GBM patients, suggesting a weak link (if any) of TCTN1 and cilia in human gliomas. Functions and molecular mechanisms of TCTN1 in glioma warrant more investigations.

Characterized by dramatic molecular and histologic heterogeneity, GBM has recently been classified into distinct subtypes with clinical relevance, opening the way for treatments to be directed at subtype-specific mechanisms [5,60]. In addition, for each molecular subtype, genetic alterations in several key genes were significantly different. The TCGA dataset offers an opportunity to investigate the relationship between gene expression, molecular subtypes and genetic alterations [61-64]. Therefore, we studied the expression preference of *TCTN1* in different subtypes and its association with genetic aberrations in the TCGA cohort. We found that *TCTN1* was dramatically decreased in the proneural subtype compared to other three subtypes, which is in concordance with the previous finding that the proneural subtype has a trend toward longer survival compared with other subtypes [5]. For common genetic alterations of GBM, *TCTN1* was expressed in correlation with 10 of them, i.e. mutations of *TP53*, *IDH1* and *ATRX*, amplifications of *EGFR*, *PDGFRA* and *MYCN*, deletions of *CDKN2A*, *CDKN2B*, *PTEN* and *PARK2*. Interestingly, for several of them (*TP53* mutation, *EGFR* amplification, *PTEN* deletion and *PARK2* deletion), the association was restricted in non-proneural or proneural subtype. For instance, within non-proneural subgroups, the status of *PTEN* deletion did not influence the levels of *TCTN1* expression. However, within the proneural subtype, patients with no CNA of *PTEN* had dramatically lower *TCTN1* expression compared to *PTEN* deleted patients. These findings provided a clue for further research of the regulation of *TCTN1* expression in GBMs.

We also investigated the relationship of *TCTN1* expression and patients’ clinical outcome stratified by different molecular subtypes and status of key genetic alterations. As a consequence, when we looked at *TCTN1* impact on survival based on molecular subtype, only the proneural and mesenchymal subtype retained significance. This analysis showed that the influence of *TCTN1* expression on survival outcome shows high subtype specificity with very strong effect in the proneural and mesenchymal subtypes and almost no effect in the other subtypes, thus the full sample analysis effectively showed a dilution of the effect in these two subtypes. In particular, patients within the proneural subtype are expected to have a slightly better prognosis compared to other subtypes [5]. However, we noted that within the proneural subgroup patients with high *TCTN1* expression suffer from especially poor prognosis than those with low *TCTN1* expression. Moreover, we also investigated status of genetic alterations in TCGA dataset and stratified the patients with GBM into two subgroups by these molecular features. Our results showed that the effect of *TCTN1* expression on patients’ survival rely on genetic background. It should be noted that, *TCTN1* could divide patients with no *PTEN* copy number change into two subsets with totally distinct outcome, although there was no difference for survival of *PTEN* deleted patients with different *TCTN1* expression, suggesting distinct effect of *TCTN1* on clinical outcome dependent on status of *PTEN* deletion. Similar results could also be observed for other several alterations, in detail, high expression of *TCTN1* could predict poor prognosis for patients with no *EGFR* change, no *PDGFRA* change, no *MYCN* change, *PARK2* deletion, *CDKN2A* deletion or *CDKN2B* deletion. However, further perspective studies are still warranted to unveil the underlying mechanisms.

Our analyses in these independent cohorts suggested a key role of *TCTN1* gene in tumorigenesis and progression of GBM, yet there has been no direct report on its function in cancer biology. Thus we performed *in vitro* experiments in two GBM cell lines through enforced expression or depletion of TCTN1. Consequently, we observed that TCTN1 overexpression evidently promoted cell proliferation, whereas TCTN1 depletion dramatically hampered cell growth. These results were consistent with the augmented expression and prognostic value of TCTN1 in GBM clinical tissues, suggesting that its survival detriment role may be in part due to the ability of the TCTN1 protein to regulate proliferation of GBM cells. Functional study in cell lines further highlighted potential therapeutic value of TCTN1 in treatment of patients with GBM, albeit the molecular mechanisms were still far from elucidation.

## Conclusions

In summary, TCTN1 was significantly elevated in human GBMs, and predicted poorer prognosis of GBM patients as a novel prognostic factor, which was found in a TMA analysis of a Chinese cohort and confirmed in two independent international cohorts. Furthermore, the expression profile and prognostic value of TNTN1 were associated with different molecular subtype and genetic alterations of GBM in analyses of the TCGA dataset. Moreover, TCTN1 played an important role in proliferation of GBM cells, suggesting its potential application as a therapeutic target for future GBM treatment.

## Additional file

Additional file 1: Figure S1. TCTN1 protein expression was analysed by immunohistochemistry staining and positive staining rate of TCTN1 in normal brain samples and GBMs was indicated as a scatter plot. *P* value was determined by Student’s t-test. **Figure S2.** *TCTN1* mRNA expression was significantly different in subgroups of GBMs in the TCGA cohort according to status of *IDH1* mutation (A), *ATRX* mutation (B), *MYCN* amplification (C) or *PDGFRA* amplification (D) as indicated. A single spot represents the *TCTN1* expression value (in log 2 scale) of an individual patient, with a line in the middle representing the mean expression value. *P* values were determined by Student’s t-test. **Figure S3.** Correlations between *TCTN1* with *GLI1*(A) and *PTCH1*(B) levels in TCGA cohort. **Figure S4.** Kaplan-Meier curves were plotted according to different *TCTN1* gene expression for overall survival of GBM patients in the TCGA cohort stratified by the status of EGFR amplification (A, B), PDGFRA amplification (C, D), MYCN amplification (E, F), PARK2 deletion (G, H), CDKN2A deletion (I, J) and CDKN2B deletion (K, L) as indicated. *P* values were obtained from log-rank test.

## Abbreviations

TCTN1: Tectonic family member 1
GBM: Glioblastioma
TMA: Tissue microarray
TCGA: The Cancer Genome Atlas
REMBRANDT: Repository for Molecular Brain Neoplasia Data
OS: Overall survival
PFS: Progression-free survival
HR: Hazard ratio
CI: Confidence interval
