# Supplementary material for: Expression and prognostic significance of TCTN1 in human glioblastoma
Source: J Transl Med. 2014 Oct 11;12:288. doi: 10.1186/s12967-014-0288-9 (PMC4198629; doi:10.1186/s12967-014-0288-9)
Supplement: Additional file 1: Figure S1. — TCTN1 protein expression was analysed by immunohistochemistry staining and positive staining rate of TCTN1 in normal brain samples and GBMs was indicated as a scatter plot. P value was determined by Student’s t-test. Figure S2. TCTN1 mRNA expression was significantly different in subgroups of GBMs in the TCGA cohort according to status of IDH1 mutation (A), ATRX mutation (B), MYCN amplification (C) or PDGFRA amplification (D) as indicated. A single spot represents the TCTN1 expression value (in log 2 scale) of an individual patient, with a line in the middle representing the mean expression value. P values were determined by Student’s t-test. Figure S3. Correlations between TCTN1 with GLI1(A) and PTCH1(B) levels in TCGA cohort. Figure S4. Kaplan-Meier curves were plotted according to different TCTN1 gene expression for overall survival of GBM patients in the TCGA cohort stratified by the status of EGFR amplification (A, B), PDGFRA amplification (C, D), MYCN amplification (E, F), PARK2 deletion (G, H), CDKN2A deletion (I, J) and CDKN2B deletion (K, L) as indicated. P values were obtained from log-rank test. [file 12967_2014_288_MOESM1_ESM.pdf]

Expression and prognostic significance of TCTN1 in human glioblastoma

Delong Meng[1*], Yuanyuan Chen[1*], Yingjie Zhao[1], Jingkun Wang[1], Dapeng Yun[1], Song Yang[2], Juxiang Chen[3], Hongyan Chen[1], Daru Lu[1 §]

[1]State Key Laboratory of Genetic Engineering and MOE Key Laboratory of Contemporary Anthropology, School of Life Sciences, Fudan University, No. 220 Handan Road, Shanghai, 200433, PR China
[2]The Eighth Department of General Surgery and Department of Pathology, the First Affiliated Hospital of Anhui Medical University, No. 218 Jixi Road, Hefei, 230022, PR China
[3]Department of Neurosurgery, Shanghai Institute of Neurosurgery, Changzheng Hospital, Second Military Medical University, No. 415 Fengyang Road, Shanghai, 200003, PR China
*These authors contributed equally to this work.
[§]Corresponding author

Additional files

Additional file 1 as PDF

Additional file 1 Figure S1. TCTN1 protein expression was analysed by immunohistochemistry staining and positive staining rate of TCTN1 in normal brain samples and GBMs was indicated as a scatter plot. *P* value was determined by Student's t-test.

Additional file 1 Figure S2. *TCTN1* mRNA expression was significantly different in subgroups of GBMs in the TCGA cohort according to status of *IDH1* mutation (A), *ATRX* mutation (B), *MYCN* amplification (C) or *PDGFRA* amplification (D) as indicated. A single spot represents the *TCTN1* expression value (in log 2 scale) of an individual patient, with a line in the middle representing the mean expression value. *P* values were determined by Student's t-test.

Additional file 1 Figure S3. Correlations between *TCTN1* with *GLI1*(A) and *PTCH1*(B) levels in TCGA cohort.

Additional file 1 Figure S4. Kaplan-Meier curves were plotted according to different *TCTN1* gene expression for overall survival of GBM patients in the TCGA cohort stratified by the status of EGFR amplification (A, B), PDGFRA amplification (C, D), MYCN amplification (E, F), PARK2 deletion (G, H), CDKN2A deletion (I, J) and CDKN2B deletion (K, L) as indicated. *P* values were obtained from log-rank test.

Figure S1

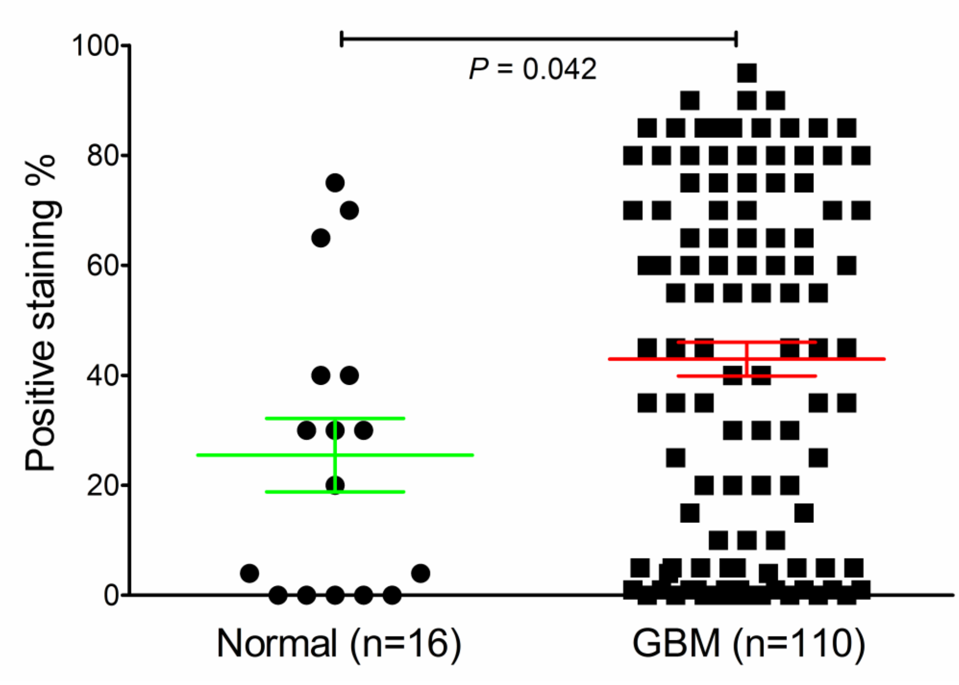

Figure S2

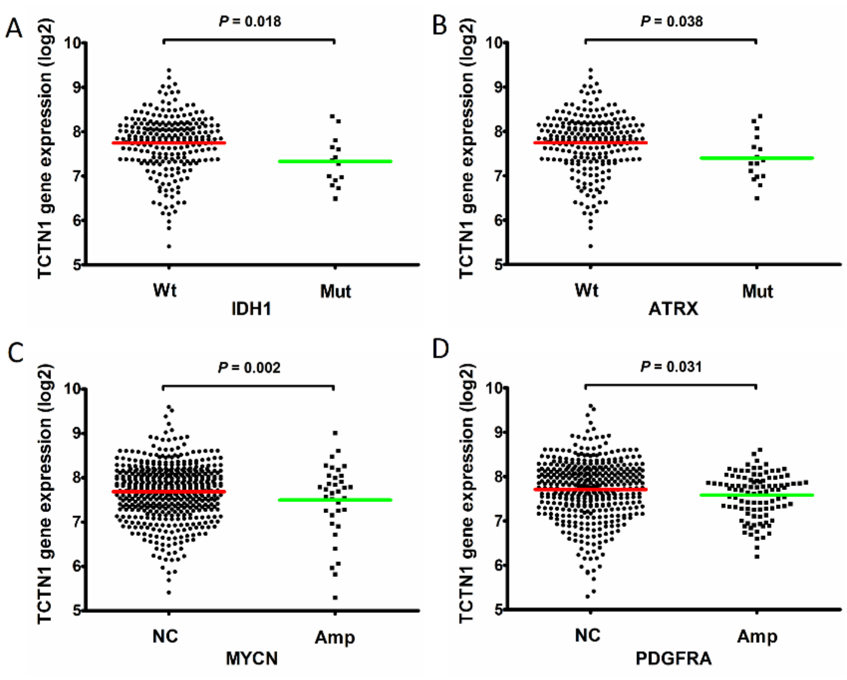
A
P = 0.018
TCTN1 gene expression (log2)
10
9
8
7
6
5
Wt
Mut
IDH1
B
P = 0.038
TCTN1 gene expression (log2)
Wt
Mut
ATRX
C
P = 0.002
TCTN1 gene expression (log2)
NC
Amp
MYCN
D
P = 0.031
TCTN1 gene expression (log2)
NC
Amp
PDGFRA

Figure S3

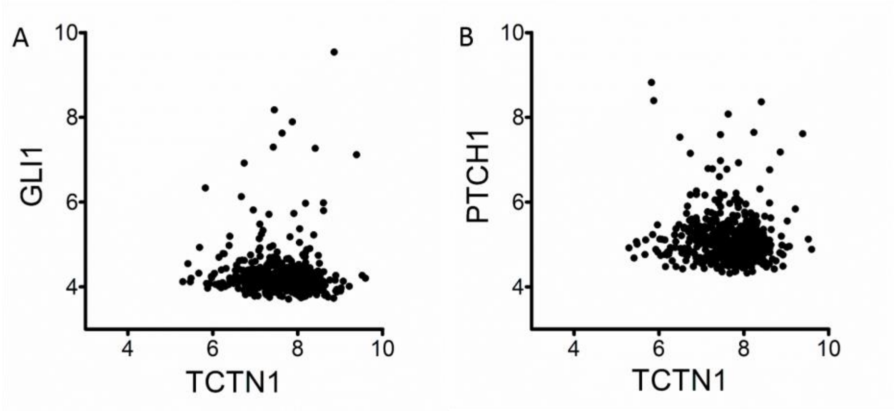

Figure S4

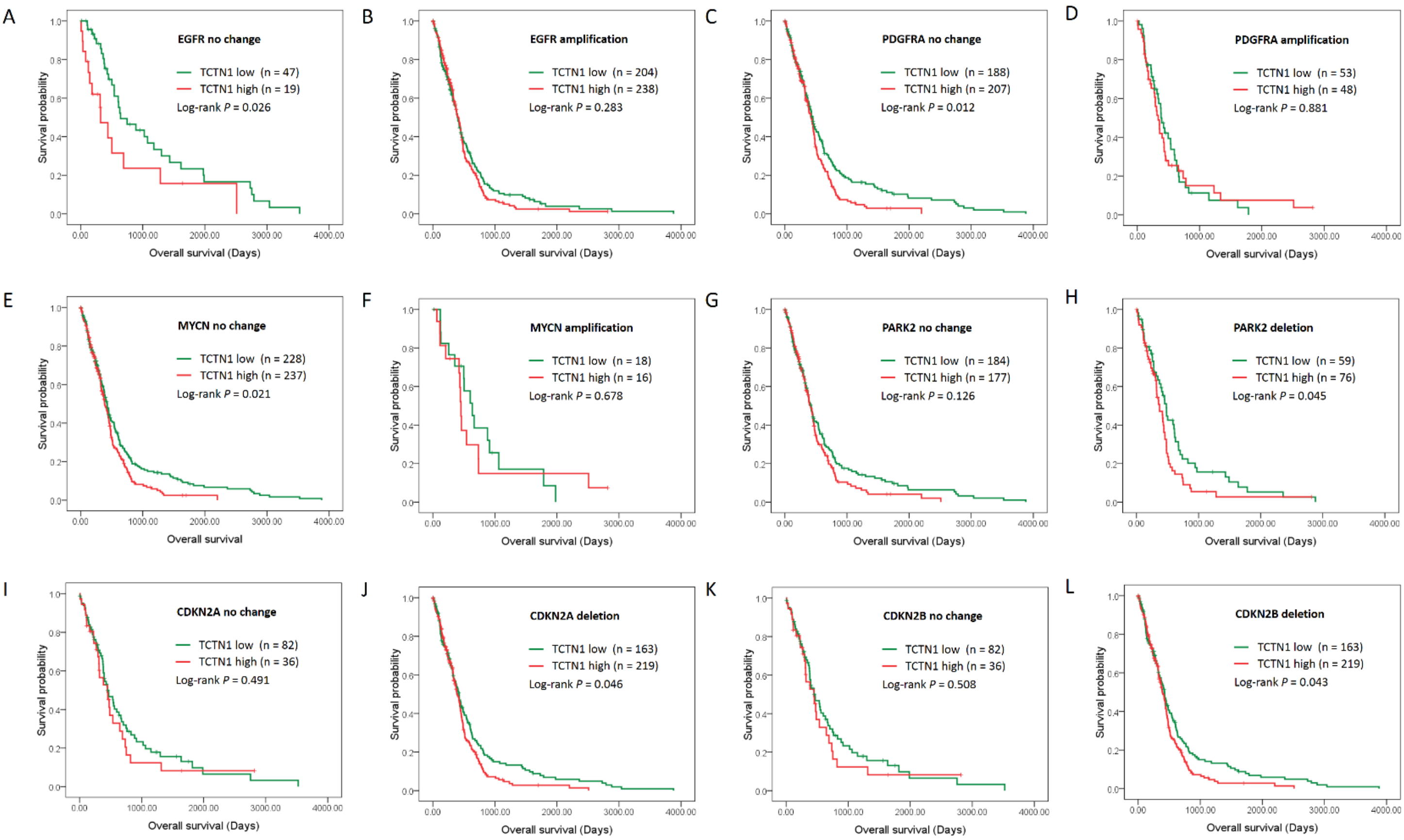
A
EGFR no change
TCTN1 low (n = 47)
TCTN1 high (n = 19)
Log-rank P = 0.026
B
EGFR amplification
TCTN1 low (n = 204)
TCTN1 high (n = 238)
Log-rank P = 0.283
C
PDGFRA no change
TCTN1 low (n = 188)
TCTN1 high (n = 207)
Log-rank P = 0.012
D
PDGFRA amplification
TCTN1 low (n = 53)
TCTN1 high (n = 48)
Log-rank P = 0.881
E
MYCN no change
TCTN1 low (n = 228)
TCTN1 high (n = 237)
Log-rank P = 0.021
F
MYCN amplification
TCTN1 low (n = 18)
TCTN1 high (n = 16)
Log-rank P = 0.678
G
PARK2 no change
TCTN1 low (n = 184)
TCTN1 high (n = 177)
Log-rank P = 0.126
H
PARK2 deletion
TCTN1 low (n = 59)
TCTN1 high (n = 76)
Log-rank P = 0.045
I
CDKN2A no change
TCTN1 low (n = 82)
TCTN1 high (n = 36)
Log-rank P = 0.491
J
CDKN2A deletion
TCTN1 low (n = 163)
TCTN1 high (n = 219)
Log-rank P = 0.046
K
CDKN2B no change
TCTN1 low (n = 82)
TCTN1 high (n = 36)
Log-rank P = 0.508
L
CDKN2B deletion
TCTN1 low (n = 163)
TCTN1 high (n = 219)
Log-rank P = 0.043
Survival probability
Overall survival (Days)
Overall survival
